# Does an Optimal Relationship Between Injury Risk and Workload Represented by the “Sweet Spot” Really Exist? An Example From Elite French Soccer Players and Pentathletes

**DOI:** 10.3389/fphys.2020.01034

**Published:** 2020-08-28

**Authors:** Adrien Sedeaud, Quentin De Larochelambert, Issa Moussa, Didier Brasse, Jean-Maxence Berrou, Stephanie Duncombe, Juliana Antero, Emmanuel Orhant, Christopher Carling, Jean-Francois Toussaint

**Affiliations:** ^1^Institut de Recherche Biomédicale et d’Épidémiologie du Sport (IRMES), Institut National du Sport, de l’Expertise et de la Performance (INSEP), Paris, France; ^2^EA7329 Institut de Recherche BioMédicale et d’Épidémiologie du Sport (IRMES), Paris, France; ^3^Fédération Française de Football, Paris, France; ^4^Fédération Française de Pentathlon Moderne, Paris, France; ^5^School of Human Movement and Nutrition Sciences, The University of Queensland, Brisbane, QLD, Australia; ^6^Centre d’Investigation en Médecine du Sport, Paris, France

**Keywords:** injury, training load, soccer (football), pentathlon, ACWR, EWMA, REDI

## Abstract

**Objective:**

To examine the relationships between the occurrence and severity of injuries using three workload ratios (ACWR, EWMA, REDI) in elite female soccer players and international male and female pentathletes.

**Materials and Methods:**

Female soccer players in the U16 to U18 national French teams (*n* = 24) and international athletes (*n* = 12, 4 women and 8 men) in the French modern pentathlon team were monitored throughout an entire season. The Acute Chronic Workload Ratio (ACWR), the Exponentially Weighted Moving Averages (EWMA), and the Robust Exponential Decreasing Index (REDI) were calculated for internal load by the ROE method in soccer and external load in pentathlon. The occurrence and severity of injuries (determined according to time-loss) were quantified in the sweet spot zone [0.8; 1.3] and compared to the other zones of load variation: [0; 0.8], [1.3; 1.5], [1.5; +8], using the three ratios.

**Results:**

Over the study period, a total of sixty-six injuries (2.75 per athlete) were reported in the soccer players and twelve in pentathletes (1 per athlete). The cumulative severity of all injuries was 788 days lost in soccer and 36 in pentathlon: respectively, 11.9 days lost per injury in soccer player and 3.0 per pentathlete. The mean values across the three methods in soccer showed a higher number of injuries detected in the [0; 0.8] workload ratio zone: 22.3 ± 6.4. They were 17.3 ± 3.5 in the sweet spot ([0.8–1.3] zone) and 17.6 ± 5.5 in the [1.5; +8] zone. In comparison to the [1.5; +8] zone, soccer players reported a higher number of days lost to injuries in the presumed sweet spot and in the [0–0.8] zone: 204.7 ± 28.7 and 275.0 ± 120.7 days, respectively. In pentathletes, ten of the twelve injuries (83.3%) occurred in the presumed sweet spot. REDI was the only method capable of tracking workloads over all-time series.

**Conclusion:**

In the present cohort of elite soccer players and pentathletes, acute chronic workload calculations showed an association with injury occurrence and severity but did not provide evidence supporting existence of a sweet spot diminishing injury risk.

## Introduction

In contemporary sport, monitoring programs typically aim to enable optimal adjustment and prescription of training volume and intensity. Information pertaining to training such as internal and external load, recovery, fatigue, tests, performance, and injuries enables the creation of datasets that allow athletes’ current states of fitness and fatigue to be tracked holistically (Robertson et al., 2016). The creation of decision support tools based on this information requires complex data transformation processes, which are currently still limited in sports science (Claudino et al., 2019). Additionally, monitoring presents challenges for practitioners as no single variable, marker, test, or tool can currently provide a complete picture of athletes’ current state of readiness or injury risk (McGuigan, 2017). Nevertheless, variations in workload are commonly analyzed in relation to performance level, session regulation, and injury emergence.

A common method for analyzing the effects of variations in workload is the Acute Chronic Workload Ratio (ACWR) (Gabbett, 2016). The ACWR describes the ratio between the workload accumulated during the last seven days (i.e., acute training load) relative to the mean workload over the last 28 days (chronic) (Blanch and Gabbett, 2016; Hulin et al., 2016). In comparison to the chronic training load, the acute load is considered to reflect the risk of injury in athletic populations (Maupin et al., 2020). One interpretation method for the ACWR is its use of an optimal workload value, or “sweet spot” (i.e., match and training load associated with the lowest risk of injury) determined between 0.8 and 1.3 (Gabbett, 2016). Research also suggests an increased risk of incurring injury when the ACWR exceeds 1.5 (Blanch and Gabbett, 2016). However, the ACWR is shown to suffer from several methodological issues (Impellizzeri et al., 2020), such as mathematical coupling problems (Lolli et al., 2019) and also lacks sensitivity (Menaspà, 2017; Williams et al., 2017). Accordingly, additional workload ratios have been created including the Exponentially Weighted Moving Averages (EWMA) (Murray et al., 2017) and more recently the Robust Exponential Decreasing Index (REDI) (Moussa et al., 2019). The EWMA is an approach that gives greater weight to the load completed in the acute phase (compared to previous days/weeks), and accounts for the decreasing nature of the effects of fitness and fatigue over time through the application of exponential decay (Williams et al., 2017). The EWMA is proposed as a more suitable measure due to its greater sensitivity (Griffin et al., 2020). The REDI was developed specifically to address sparse data and discretization processes. Indeed, the REDI can be calculated with up to 30% of missing data and does not discretize or lose information by averaging the charges over a week or a month (Moussa et al., 2019). Further work is nevertheless warranted to explore its utilization in elite athletic populations.

However, these ratios still form only one of many variables that are potentially associated with performance or injury occurrence. Irrespective of methodology, using a single workload ratio can be considered an overly simplistic method for determining the optimal training load or overall injury risk. As such, concerns have been expressed regarding injury prediction when using these ratios (Hulme et al., 2019; Wang et al., 2020). Based only on these ratios, the sweet spot method proposed is similar to an isolated variable approach, which hinders monitoring processes and consequently good practice in the field of sport science (Haugen, 2019). The data volume and quality, the model used, and various false positive issues can lead to difficulties in the implementation of injury prediction. Furthermore, while load variations can be dangerous if they are illogically applied or excessive, they are also necessary to impact athlete adaptation. Consequently, it is important that workload is monitored and controlled from an individualistic standpoint. While, the ratio alone cannot provide a holistic perspective on injury prevention, it can still provide valuable insights into training load. Indeed, the relationships between workload ratios and injury occurrence and severity using a collective and individual analysis can provide further insight into the existence of the sweet spot.

Despite these limitations, a substantial number of investigations in team sports have nevertheless employed such ratios. However, only a limited number of studies have explored the impact of ACWR in monitoring the training practices of elite female athletes (Collette et al., 2018; Paulauskas et al., 2019). Although the injury rate in top-level female soccer players is high, especially in the U19 category (Junge and Dvorak, 2007), there are to the present authors’ knowledge, no studies that have directly examined workload monitoring (comparisons using ACWR, EWMA and REDI ratios) in female soccer players. Similarly, the literature on individual sports is more limited and to our knowledge no studies have investigated the relationship between workload and occurrence or severity in elite pentathletes. Finally, only a small number of investigations have examined the relationship between the ACWR and injury severity (Bowen et al., 2017, 2019; Enright et al., 2020). Additional work utilizing current workload methods to better understand injury severity in elite soccer female athletes and pentathletes is thereby warranted.

The purpose of this study is to examine the relationships between the occurrence and severity of injuries using three workload ratios (ACWR, EWMA, REDI) in elite female soccer players and international male and female pentathletes.

## Materials and Methods

### Sample and Study Design

Workload and injuries were prospectively monitored in high-level female athletes in the French under 16 to under 18 years old national soccer teams and in international male and female modern pentathletes for the complete duration of their respective seasons.

### Soccer

Data were collected from 24 female players (17.1 ± 0.8 years), who were part of the U16 to U18 French national teams during the entirety of the 2016/2017 season. These age categories were habitually grouped together for training. The Rating of Observation Exertion (ROE) was collected as the workload metric for all training sessions. As previously recommended, the coach gave his rating of observed exertion for each individual player within a 30 min interval following each training session (Doeven et al., 2017). Training loads, typically reported as arbitrary units (AU), were calculated by multiplying each player’s training time (minutes) by session-ROE.

### Pentathlon

Data were collected from 12 elite athletes (4 women and 8 men) belonging to the French modern pentathlon team during the 2018/2019 season. The mean ages for female and male athletes were 23.5 ± 1.3 and 24.7 ± 2.7 years, respectively. During this period, external workload was quantified individually across the training disciplines using a smartphone application developed for this purpose. External training load indicators included the total kilometers swum, run, and accumulated in the combined laser-run event, in addition to the number of touches in the fencing event.

Pentathletes were included for two reasons:

1. To be able to analyze the external load in addition to the internal load.
2. To determine the effects of analyzing a dataset with missing data in order to test the robustness of the different ratios (ACWR versus EWMA versus REDI).

### Training Load

For soccer, the internal load, characterized by the session-ROE was used to calculate the three ratios (Doeven et al., 2017). For pentathlon, the external training loads for each discipline were standardized according to a distribution between 0 and 1:

$$\mathrm{Standardized}\mathrm{training}{\mathrm{load}}_{i,j}=$$

$$\frac{\mathrm{Training}{\mathrm{load}}_{i,j}-\min\left(\mathrm{Training}{\mathrm{load}}_{j}\right)}{\max\left(\mathrm{Training}{\mathrm{load}}_{j}\right)-\min\left(\mathrm{Training}{\mathrm{load}}_{j}\right)}$$

i  :day ; j  :discipline

### Injuries

An injury was defined as any event resulting in a cessation of at least one training session or competition. Injuries were self-reported by athletes and cross-checked with the medical staff. Various criteria exist for the measurement of severity, but the most widely used in sports medicine is the duration of time lost (Bahr et al., 2020). Previous consensus statements highlighted the simplicity of implementing this method and the potential for collection by non-medical doctors, especially in soccer (Fuller et al., 2006), track and field (Timpka et al., 2014) and swimming (Mountjoy et al., 2016). The severity of injuries was recorded according to the time lost, defined as the number of days that the athlete was unavailable for training and competition, from the date of onset until the athlete had resumed training and competition (Bahr et al., 2020). Time loss due to illness episodes was not included.

### Research Ethics and Data Security

Prior to participation, athletes were informed about the purpose of the study and the data collection involved and written consent was obtained. For athletes below the age of 18 years, written consent was provided by their parent/guardian. All investigations conformed to the code of ethics of the World Medical Association (Declaration of Helsinki) and were approved by the Institutional Ethics Committee. Data collection was compliant with the General Data Protection Regulations applied in the European Union.

### Data Analysis

For both sports, three training load calculations were applied to each individual:

– The ACWR (Gabbett, 2016) was calculated using rolling averages, with the previous 7 days defined as the acute load and the previous 28 days as the chronic load. The average of the acute (7-day) period was divided by the average of the chronic (28-day) period.
– The EWMA (Williams et al., 2017) was calculated using the equation:
  $${\text{EWMA}}_{\text{today}}={\text{Load}}_{\text{today}}\cdot \lambda_{a}+$$
  $$\left(\left(1-\lambda_{a}\right)\cdot {\text{EWMA}}_{\text{yesterday}}\right)$$

where λ_*a*_ is a value between 0 and 1 that represents the level by which the workload decreases. It is defined as:

$$\lambda_{a}=\frac{2}{N+1}$$

N:timedecayconstant.

1. The REDI (Moussa et al., 2019) was calculated using the equation:
  $${\text{REDI}}_{\text{today}}^{\lambda }=\frac{1}{\sum_{i=0}^{N}\alpha_{i}^{\lambda }}\sum_{i=0}^{N}\alpha_{i}^{\lambda }.WL_{i}$$
  $$\text{And}\alpha_{i}^{\lambda }=\{\begin{array}{cc} 0 & \mathrm{if}{\mathrm{WL}}_{\text{i}}\mathrm{is}\mathrm{missing}\\ e^{-\lambda i} & \text{otherwise} \end{array}$$

where:

• *WL*_*i*_ is the workload of the past *i*th day before the current day.
• *N* is the total number of previous days in our dataset.
• λ is a parameter that can be adjusted in order to decrease the weighting of the workload.

In order to compare the three training load calculations, the EWMA and the REDI were expressed as the ratio of acute load to chronic load as done in the ACWR. The acute load was calculated using EWMA by setting *N* = 7 (lambda for the REDI = 0.25) and the chronic load by setting *N* = 28 (lambda for the REDI = 0.07) (Cousins et al., 2019). The same lambda coefficients were used for the calculation of the EWMA and REDI ratio of acute load to chronic load.

For each ratio, three indicators were created and observed with regards to four different zones: [0; 0.8], [0.8; 1.3], [1.3; 1.5], [1.5; +∞]. These indicators were (1) the number of injuries, (2) the injury severity (days of absence due to injury), and (3) the injury severity related to the time spent (days) in the different zones. For each ratio, three χ^2^ tests were used to assess inequalities in the proportion of injury occurrences and severity in each of the zones ([0; 0.8], [0.8; 1.3], [1.3; 1.5], [1.5; +∞]):

– a test of adequacy to a uniform law ($\forall i\in \left\{z1,z2,z3,z4\right\},P\left(X=x_{i}\right)=\frac{1}{4}$) was conducted on the number of injuries occurring in each zone.
– a test of adequacy to a uniform law ($\forall i\in \left\{z1,z2,z3,z4\right\},P\left(X=x_{i}\right)=\frac{1}{4}$) was conducted on the number of injury-free days occurring in each zone.
– a test of adequacy to a probability law *P*(*X* = *x*_*i*_) = *p*_*i*_, where *p*_*i*_ represents the proportion of days spent in each of the zones, was conducted on the number of days absent owing to injuries occurring in each zone.

The significance level of the chi-squared test was set at the threshold α = 0.05 and the residuals from this test were compared to the quantile of the normal law *z*_0.05/2_ = −1.96 and $z_{1-\left(\frac{0.05}{2}\right)}=1.96$.

All statistical analyses were performed using R (version 3.3.2; The R Foundation for Statistical Computing, Vienna, Austria).

## Results

**FIGURE 1:**
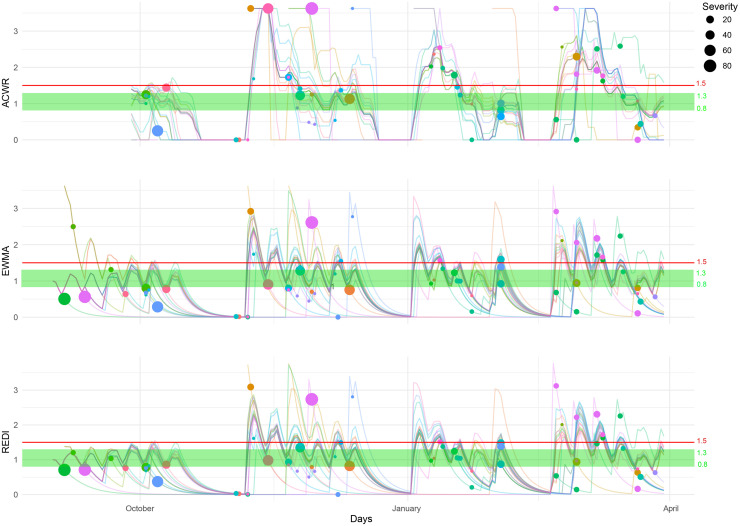
Individual training load plotted with the ACWR, EWMA and REDI indices, and the occurrence and severity (days) of injuries for elite U16–U18 female soccer players. Each line represents an athlete’s training load over time calculated by ACWR (top chart), EWMA (middle chart) and REDI (bottom chart). Each dot represents an injury and the width of the dot depicts the severity. The green zone represents the so-called “sweet spot” for the three ratios, the potentially optimal workload ratio value between 0.8 and 1.3. Above the red line are the ratio values greater than 1.5.

**FIGURE 2:**
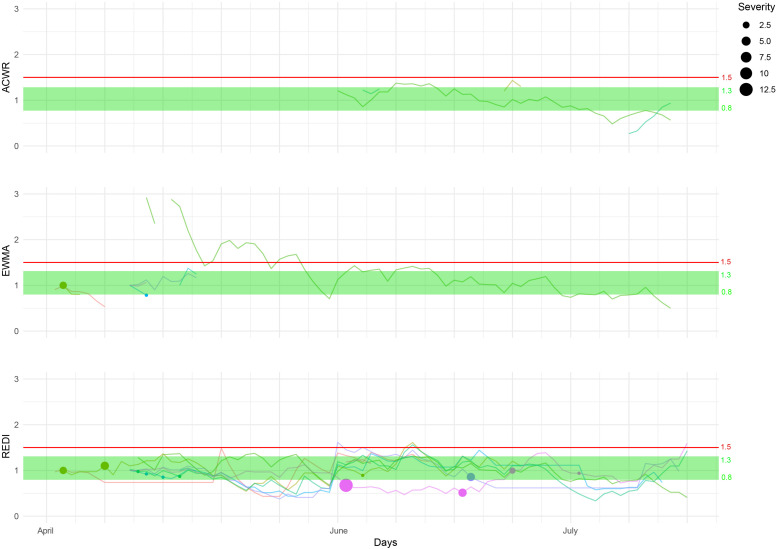
Individual training load plotted with the ACWR, EWMA, and REDI indices, and the occurrence and severity (in days) of injuries in the French national pentathlon team. Each line represents an athlete training load follow-up calculated by ACWR (top chart), EWMA (middle chart) and REDI (bottom chart). Each dot represents an injury, the width of the dot depicts the severity. The green zone represents the so-called “sweet spot,” the potentially optimal workload ratio value between 0.8 and 1.3. Above the red line are the ratio values greater than 1.5.

The results presented in Figures 1, 2 demonstrate the relationships between various training loads, represented by ACWR, EWMA, and REDI, and the occurrence and severity of injuries in soccer and modern pentathlon athletes.

### Injury Rates in Soccer

Sixty-six injuries in total were reported over the study period resulting in an average of 2.75 injuries per player. Figure 1 presents individual training load plotted with the ACWR, EWMA and REDI indices, and the occurrence and severity (days) of injuries.

For all workload ratios combined, the mean number of injuries in the [0; 0.8] zone was 22.3 which was significantly higher than in the other zones (Table 1). Altogether, a mean of 17.3 injuries were reported in the zone between [0.8; 1.3] and 17.7 for ratios in the [1.5; +∞] zone. For the ACWR, only 57 of 66 injuries were computable with this ratio; 15 injuries appeared in the [0; 0.8] zone, 14 in the [0.8; 1.3] zone, and 23 in the [1.5; +∞] zone, the latter being significantly higher than the other two (Table 1).

**TABLE 1:** Number and severity of injuries by ratio calculation and zone in elite female soccer players.

Area	ACWR	EWMA	REDI	Mean ± SD
**Numbers of injuries**				
<0.8	15	27^†^	25^#^	22.3 ± 6.4**
0.8–1.3	14	17	21	17.3 ± 3.5
1.3–1.5	5	4	8	8.9 ± 2.1
>1.5	23*	18	12	17.6 ± 5.5
Total	57	66	66	−
**Severity of injuries**				
<0.8	137	361^†^	327^#^	275 ± 120.7**
0.8–1.3	172	216	226^#^	204.7 ± 28.7**
1.3–1.5	36	29	94	53 ± 35.7
>1.5	254*	182	141	192.3 ± 57.2
Total	599	788	788	−
**Severity related to time spent (in days) in zones**				
<0.8	0.08	0.15	0.15	0.13 ± 0.04**
0.8–1.3	0.19*	0.16	0.15	0.17 ± 0.02
1.3–1.5	0.12	0.07	0.21^#^	0.13 ± 35.7
>1.5	0.22*	0.24^†^	0.19^#^	0.22 ± 0.03**

For each ratio, the [1.3; 1.5] zone had significantly less injuries and injury severity was lower. *Significantly higher by zone for the ACWR; ^†^significantly higher by zone for the EWMA; ^#^significantly higher by zone for the REDI; **significant differences for the average of the ratios.

Regarding EWMA, 27 injuries appeared in the [0; 0.8] zone. This frequency was significantly higher than the 17 injuries in the [0.8; 1.3] zone, and the 18 injuries in [1.5; +∞] zone (Table 1). For the REDI, 25 injuries occurred in the [0; 0.8] zone. This result was significantly higher than the 21 injuries in the [0.8; 1.3] range and the 12 injuries in the [1.5; +∞] zone (Table 1). No significant differences were observed between the [0.8; 1.3] and [1.5; +∞] zones.

### Severity of Injury in Soccer

The cumulative time layoff for all injuries across the seasons was 788 days, resulting in an average of 11.9 days lost per injury. Across the three methods, the average severity per zone was 275 days lost in the [0; 0.8] zone versus 204.7 days in the zone between [0.8; 1.3], and 192.3 days in the zone above 1.5 (Table 1). The [0; 0.8] and [0.8; 1.3] zones presented a higher number of days lost (Table 1). For the ACWR, the cumulative length of time off was 137 days in the [0; 0.8] zone versus 172 days in the [0.8; 1.3] zone and 254 days in the [1.5; +∞] zone, which was significantly higher than the lower 2 zones. For the EWMA, 361 days were cumulatively missed in the [0; 0.8] zone. This total was significantly higher than the 216 days missed in the [0.8; 1.3] zone and the 182 days layoff in the [1.5; +∞] zone. For the REDI, injuries resulted in a cumulative 327 days layoff in the [0; 0.8] zone and 226 days layoff in the [0.8; 1.3] zone. The probability of being absent due to an injury was significantly higher in these two zones compared to the [1.5; +∞] zone, where the cumulative days off was 141 days (Table 1).

### Severity Related to Time Spent (Days) in Zones

When severity was related to the time spent in each zone, average scores across all methods were 0.13 in the [0; 0.8] zone versus 0.17 in the zone between [0.8; 1.3], and 0.22 for ratios in the zone >1.5 (Table 1). There was a significantly greater chance of being absent due to injury in the zone >1.5. For the ACWR calculation, there was a significantly greater chance of being absent due to injury in the zone between [0.8; 1.3] and the >1.5 zone. The severity related to time spent using the EWMA calculation was significantly greater in the zone for ratios >1.5 (0.24). Scores obtained for REDI were 0.15 in the [0; 0.8] zone, 0.15 in the zone between [0.8; 1.3], and 0.19 for ratios in the zone >1.5. There was a significantly greater chance of being absent due to injury in the zone with the highest ratio compared to the other zones (Table 1).

### Injuries in Pentathlon

In the modern pentathletes, only the REDI calculation (method robust to 30% of missing data) was able to provide continuity in the monitoring (Figure 2). The proportion of missing data in the pentathlon follow-up was 25.4%. The number of injuries throughout the study duration totaled 12 (resulting in an average of 1.0 injuries per athlete). Ten injuries among the 12 were observed in the “sweet spot” range (the [0.8; 1.3] zone). The cumulative severity of all injuries was 19 days for injuries that occurred within the range between 0.8–1.3 and 17 days for the other zones, resulting in an average of 3.0 days lost per injury.

## Discussion

The main finding of the present study is that workloads within the suggested sweet spot zone were not associated with a lower injury rate in elite female soccer players or pentathletes. The greatest number of days lost to injury among female soccer players actually occurred within the sweet spot range and in the [0; 0.8] zone. The only method that provided a ratio able to monitor workload and injury occurrence among pentathletes was the REDI. For this ratio, injuries mainly occurred in the sweet spot.

### Workloads Ratios and Soccer

To our knowledge, the current study is the first to examine session-ROE association with injury occurrence and severity. In elite soccer, the monitoring of workloads informs training and preparation programming to help maximize adaption and minimize injury risk. Three recognized methods were employed to investigate whether injury occurrence was associated with workload. In contrast to earlier work using the ACWR technique, no evidence was found to support a U-shaped relationship between ACWR values and subsequent injury likelihood (Blanch and Gabbett, 2016). This difference might exist owing to Blanch and Gabbett’s work being based upon aggregated categorical data from a series of research investigations (Blanch and Gabbett, 2016) and not upon follow-up data of the same athletes over time (Cousins et al., 2019). Another possible explanation may be that in this study ROE was used to calculate ratios whereas Blanch and Gabbett used total running load data from rugby league players and total running load and high-speed running loads from the Australian football player’s among others. In the present study, an arguably more suitable method for continuous data (the REDI) was used (Moussa et al., 2019) with results showing the opposite relationship; more severe injuries occurred when workload was within the proposed sweet spot. In addition, almost a third (27.5%) of the total injuries (and more than 83% in the pentathletes – discussed below) occurred within the sweet spot. This finding is not in agreement with results from an investigation in professional soccer players in which a lower risk of injury was observed for ACWR values between 1.00 and 1.25 (Jaspers et al., 2018). Jaspers et al. used total distance covered, distance covered at high speed, number of accelerations, decelerations, and RPE-session as parameters for ACWR calculations. However, more recent work (Enright et al., 2020) tends to support our findings: 53% of injuries reported in elite male soccer players occurred in the sweet spot. Another previous study also observed poor injury prediction from ACWR ratios in soccer (Fanchini et al., 2018). These findings generally reinforce the idea that with current monitoring methods, no relationship exists between sweet spot values and injury risk. This could be linked to discretization processes that are induced by a calculation based on weekly and monthly averages and that result in information loss, false discovery rates and less accurate estimates than continuous models (Carey et al., 2018).

Whilst disagreement exists around the pertinence of the sweet spot, evidence nevertheless suggests that critical ACWR thresholds should not be exceeded to avoid an increase in injury risk (Hulin et al., 2014, 2016; Bowen et al., 2019). The present study adds further confirmation to this as the number of injuries and the severity related to time spent in each zone showed a higher risk in the [1.5; +∞] zone for the ACWR ratio. These results are potentially affected by the existing data discretization bias caused by these ratios (Wang et al., 2020). While thresholds are a useful initial step in minimizing injury risk in response to a training program, such approaches oversimplify the relationship between loads and injuries as they rely on basic associations. Specific thresholds, and the consequential sweet spot, fail to consider the non-linear relationships between variables or differences between individuals. Indeed, the relationship between workload and injuries is complex, recursive, and individualistic, with varying levels of influence among variables as they interact in a non-linear way (Bittencourt et al., 2016; Roe et al., 2017).

### Workloads Ratios and Modern Pentathlon

To our knowledge this study is the first to explore the relationship between workload variations and injuries in modern pentathlon; three key findings are reported. The first is the low frequency of injuries over the study period (an average of 1.0 injury per player per season). This is similar to what was shown in a previous study on world class modern pentathlon athletes, where only two incidents per athlete were reported during a season (Kelm et al., 2003). The authors interviewed athletes about their individual training practices and sports related injuries, muscle damage, and illnesses. Their results highlighted that that two pentathlon events, running and swimming, were the ones most frequently linked to muscle damage, and disease (Kelm et al., 2003). Therefore, it seems relevant to further pursue research focusing on the relationship between training loads and injuries in this sport and its multiple events using larger datasets. The small number of injuries observed in the present study could be explained by the definition used, which requires time-loss. This definition does not take into account periods where athletes are still training (e.g., in shooting) while injured (e.g., in a leg) (Bahr et al., 2020). In pentathlon, the multidisciplinary nature of the sport makes it possible to continue training despite injuries. The second key finding concerns the distribution of injuries, with 10 of the 12 injuries occurring in the sweet spot. This suggests that for this sport, a ratio approach might not be applicable for estimating the risk of injury. Finally, it is important to mention that owing to missing data over the study period, the construction of a robust and sustainable workload monitoring tool was only achieved using the REDI approach.

### Severity of Injuries in Soccer and Pentathletes

It is necessary to consider injury severity since the duration athletes are absent from training or competition has a negative impact on individual and team performances (Carling et al., 2015). Currently, few studies have examined the relationship between the ACWR and injury severity (Bowen et al., 2017, 2019; Enright et al., 2020). Those that have previously focused on this relationship classified severity into 4 categories (minimal, mild, moderate, and severe), thereby limiting the feasibility to examine the effect of workload on injury severity (Enright et al., 2020). For example, a paper using this approach to study 8 teams competing in UEFA professional soccer leagues across 2 seasons reported that none of the ACWRs or accumulated weekly loads were associated with the number of days a player missed through injury (Enright et al., 2020). Therefore, coupling both the frequency and severity of injuries is important as it enables researchers to discern not only the occurrence of injuries, but more importantly, those that prevent athletes from training or competition for an extended time period. This integrative perspective is central to an injury prevention program. New information is also provided here when the number of days lost is reported by both absolute (number of absent days due to injury) and relative severity (time spent in the different zones). A greater absolute severity was observed in the sweet spot and a greater relative severity was reported in high load ratios.

### Workload Ratio Approach

The present study highlights that a moving average approach (EWMA) and a decreasing weight approach (REDI) offer additional information and continuity in monitoring the relationship between workload and injury compared to the ratio (ACWR) approach. Indeed, the latter demonstrates limitations concerning its monitoring consistency (Buchheit, 2016). This result is supported by studies that suggest that the ACWR approach lacks sensitivity (Menaspà, 2017; Williams et al., 2017) and suffers from mathematical coupling problems (Lolli et al., 2019). The EWMA is considered more sensitive than the ACWR ratio (Murray et al., 2017), and the REDI adds additional value as it minimizes the potential loss of data and information created by discretisation, which may introduce bias. Furthermore, these isolated ratios do not explain the entire injury phenomenon and must be related to the absolute and cumulative training loads, which provide new insights. Adapting the EWMA and REDI into ratio calculations may introduce noise as the training load is considered twice. This is however, necessary to enable comparison of the three approaches. In the future, moving average and decreasing weights approaches should be used for the purpose they were designed for. Arguably, it would also be preferable to refrain from discretizing data through the creation of ratios, and to instead use the EWMA and REDI as initially constructed to account for the loss of information on acute and chronic loads.

### Underlying Relationship Between Workload and Injuries

The emergence of an injury is a complex phenomenon (Bittencourt et al., 2016) involving a large number of parameters (performance, workload, physiology, sleep, fatigue & recovery, psychology, lifestyle, etc.). From a holistic perspective, computational methods may help in modeling the complex systems of sport injury risk (Hulme et al., 2019). Relationships between workload and injuries are recursive and individualistic, with variables interacting in a non-linear way and with differing levels of impact. For example, physiological mechanisms underlying the relationship between RPE, covered distance, mode of exercise and injuries are mediated by level of fitness and fatigue among others and this relation at t are different for the same athlete at another moment. Consequently, small variations can generate large effects as shown in complex systems. Abrupt changes without adequate adaptation to maintain equilibrium can result in a tipping point or system failure (Hulme et al., 2019).

### Limitations

The current study has several limitations. First injury severity was defined according to time-lost, which can be “blurred” due to the unclear demarcation between the end of the time-lost and the resumption of training or competition (Bahr et al., 2020). Athletes can return to competition before they have fully recovered from an injury or illness; consequently, such a definition might underestimate severity. Additionally, in some cases, the time lost does not immediately follow the occurrence of an injury and can be delayed or intermittent. Second, the lack of differentiation between contact and non-contact injuries in this study generates another bias. Studying both at the same time allows a global vision of injury occurrences, but including contact injuries could reduce the association of the ACWR or other ratios with injury risk (Bowen et al., 2019), and possibly reduce their sensitivity. Another limitation lies in the lack of distinction between the type and location of injuries. The workload ratio could be different prior to a muscular injury compared with a tendon or ligament injury (Enright et al., 2020). Fourth, no information regarding athletes’ injury or practice history are available. Fifth, the quantification of training load by ROE in soccer and regrouping external load by a standardization across the different pentathlon disciplines can be questioned. Data available in the literature on the same age category and sport (soccer) confirm a mismatch in perception of session intensity between coach and the athlete (Scantlebury et al., 2018; Vaquera et al., 2018). However, in the present study, no differences were found between soccer players’ RPE and the ROE values across a preliminary 3 month comparison (personal data). Additional work using external measures of workload is nevertheless merited.

### Practical Applications of the Study

The main practical recommendation is that practitioners should be cautious about using the ACWR to investigate potential associations between workload and injury occurrence. The second recommendation is to test and compare the REDI approach, particularly when confronted with missing workload data.

## Conclusion

This study sheds light on the relationships between acute chronic workload ratios and injury occurrence and severity. It does not demonstrate any relation between a so-called “sweet spot” and injury occurrence. In fact, the greatest severity of injuries in soccer appeared in what has been considered the sweet spot; this zone also showed the highest number of injuries for pentathletes. Consequently, decisions on workload at the individual level based on these ratios should be made with great caution. Future work will need to focus on associations between multiple parameters (workload, injury history, sleep, fatigue, recovery, etc.) in order to identify consistent indices and individual patterns to reduce injury risk.

## Data Availability Statement

The raw data supporting the conclusions of this article will be made available by the authors, without undue reservation.

## Ethics Statement

The studies involving human participants were reviewed and approved by INSEP Scientific and Medical Committee. Written informed consent to participate in this study was provided by the participants’ legal guardian/next of kin.

## Author Contributions

AS, QD, IM, SD, JA, EO, CC, and J-FT conceived, designed, performed, and analyzed the research. DB and J-MB conceived, designed, and collected data during training. AS, CC, and IM wrote the manuscript. All authors read, reviewed and approved the final manuscript.

## Conflict of Interest

The authors declare that the research was conducted in the absence of any commercial or financial relationships that could be construed as a potential conflict of interest.
